# ScyNet: Visualizing interactions in community metabolic models

**DOI:** 10.1093/bioadv/vbae104

**Published:** 2024-07-17

**Authors:** Michael Predl, Kilian Gandolf, Michael Hofer, Thomas Rattei

**Affiliations:** Centre for Microbiology and Environmental Systems Science, University of Vienna, Vienna 1030, Austria; Doctoral School in Microbiology and Environmental Science, University of Vienna, Vienna 1030, Austria; Centre for Microbiology and Environmental Systems Science, University of Vienna, Vienna 1030, Austria; Centre for Microbiology and Environmental Systems Science, University of Vienna, Vienna 1030, Austria; Centre for Microbiology and Environmental Systems Science, University of Vienna, Vienna 1030, Austria; Doctoral School in Microbiology and Environmental Science, University of Vienna, Vienna 1030, Austria

## Abstract

**Motivation:**

Genome-scale community metabolic models are used to gain mechanistic insights into interactions between community members. However, existing tools for visualizing metabolic models only cater to the needs of single organism models.

**Results:**

ScyNet is a Cytoscape app for visualizing community metabolic models, generating networks with reduced complexity by focusing on interactions between community members. ScyNet can incorporate the state of a metabolic model via fluxes or flux ranges, which is shown in a previously published simplified cystic fibrosis airway community model.

**Availability and implementation:**

ScyNet is freely available under an MIT licence and can be retrieved via the Cytoscape App Store (apps.cytoscape.org/apps/scynet). The source code is available at Github (github.com/univieCUBE/ScyNet).

## 1 Introduction

Interactions play an important role in shaping the structure and properties of microbial communities (Mataigne *et al.* 2021). They can range from beneficial to detrimental for the community members and include various forms from direct interactions, like cross-feeding, to complex traits, like biofilm formation (Zuñiga *et al.* 2017). Due to these interactions, communities of different taxa have emergent properties beyond the sum of their members’ capabilities, and are actively researched in diverse areas such as microbial ecology, medicine and biotechnology (Heinken *et al.* 2021). In recent years, the field of metabolic modelling has expanded from single organisms to communities, with the aim to gain mechanistic insights. To date, metabolic modelling has been applied to a variety of communities, including synthetic communities, human and environmental microbiomes, with community members spanning all domains of life (Heinken *et al.* 2021, Ibrahim *et al.* 2021, Saa *et al.* 2022).

Visualization of metabolic models allows communicating concepts and results. However, metabolic models, especially genome-scale ones, are highly complex as they consist of thousands of metabolites and reactions. Community metabolic models only exacerbate this problem (Ibrahim *et al.*, 2021). Many tools have been developed for the visualization of single organism models trying to deal with this complexity (König *et al.* 2012, Noronha *et al.* 2017, Hari and Lobo 2020, Pfau *et al.* 2021). One notable mention is cy3sbml (König *et al.* 2012), an app for Cytoscape, the widely used network data visualization platform (Shannon *et al.* 2003), as it converts metabolic models and all their information into a network, which can be further analysed using functions of Cytoscape or other apps. To avoid complex figures, a common technique is to display only part of the network, e.g. highlighting a single pathway as done by IDARE2 (Pfau *et al.* 2021) or showing only the immediate neighbourhood of a reaction or metabolite as done by Fluxer (Hari and Lobo 2020). For community metabolic models these approaches are not well suited, as they focus only on a local area, missing the context of the community. Instead, a visualization of the interactions would capture the essence of a community metabolic model while also drastically reducing the complexity of the figure. To our knowledge, no tool exists so far, which allows for automated visualization of interactions between community members from metabolic models.

We present ScyNet, a Cytoscape app for visualizing the exchange metabolite network of community metabolic models and the interactions between community members. ScyNet reduces the metabolic network of the whole community to community members and their consumed and produced metabolites, drastically decreasing the complexity. Flux data from flux balance analysis (FBA) and flux ranges from flux variability analysis (FVA) can be used to contextualize the visualization. ScyNet is accompanied by PyCoMo (Predl *et al.* 2024), a python package for automated generation of a community metabolic model from single organism models, which can readily be used as input. Together, they allow for quick visualization and analysis of microbial communities and their interactions starting with only the metabolic models of their members.

## 2 Description

**Figure 1.**
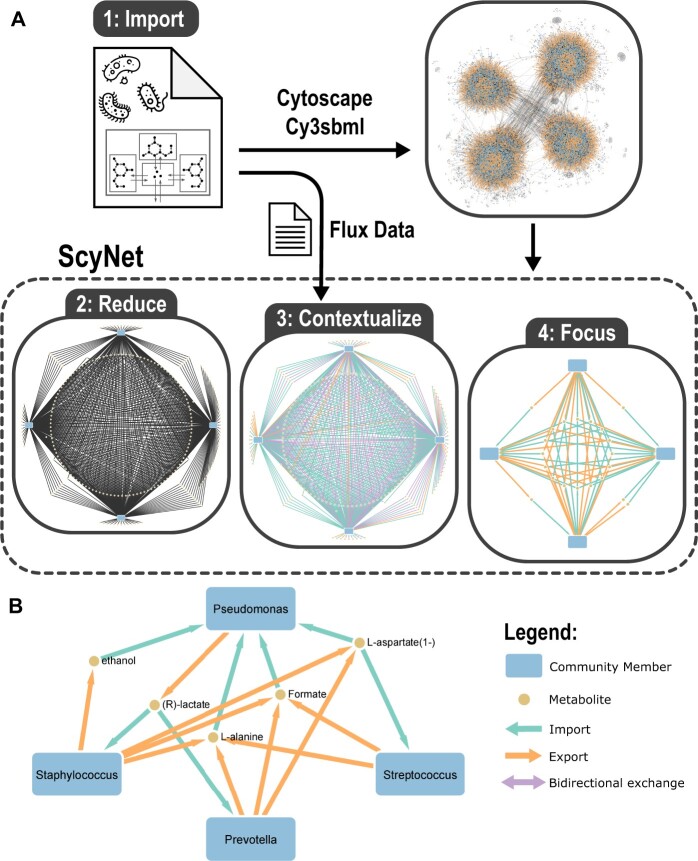
ScyNet workflow and example visualization. All networks are based on the same four AGORA models (Magnúsdóttir *et al.* 2017), converted into a community metabolic model using PyCoMo (Predl *et al.* 2024). The community members were chosen as representatives of their respective genus in a cystic fibrosis airways community model, by Henson *et al.* (2019). (A) ScyNet workflow: (Step 1) The complete network of the community metabolic model, as imported by cy3sbml (König *et al.* 2012). (Step 2) Function ‘Create Simplified Community Network’: The network after reduction by ScyNet, containing only community members, consumed and produced metabolites as nodes, and exchange reactions as edges. (Step 3) Function ‘Contextualize with Flux Data’: Visualization of flux ranges of potential exchange reactions, independent of the community composition and calculated with PyCoMo. All reactions with non-zero flux are shown. (Step 4) Function ‘Toggle Non-Cross-Fed Metabolite Visibility’: Further reduction of the network showing only cross-feeding interactions realized in a flux state found by FBA. (B) Example visualization: Replication of figure 8D by Henson *et al.* (2019): Visualization of the five metabolites identified by Henson et al. as most often cross-fed in simulations with randomly permuted exchange reaction flux bounds of samples with high abundance of *Pseudomonas*.

ScyNet is a Java app developed for Cytoscape 3.9.0 or higher (Shannon *et al.* 2003). The input is a compartmentalized community metabolic model in SBML format, such as generated by PyCoMo (Predl *et al.* 2024). To distinguish different organisms in the community metabolic model, two requirements need to be met by the input SBML file: (i) The inclusion of the name of the community member as prefix in its metabolite and compartment IDs (e.g. ‘memberId_metaboliteId’ and ‘memberId_compartmentId’) and (ii) the presence of a shared external compartment between the community members called ‘medium’. The ID of the shared external compartment can be set to a name other than ‘medium’, by specifying the ID in the SBML parameter ‘shared_compartment_id’. Additionally, reactions transporting metabolites to and from the shared external compartment are assumed to carry only a single metabolite. The ScyNet visualization workflow is started with the import of the community metabolic model in Cytoscape, which is handled by Cy3sbml (König *et al.* 2012) (Figure 1A: step 1). At step 2, ScyNet reduces the resulting network to its community members and their consumed and produced metabolites as follows. First, the nodes for each community member are generated by scanning the model for members in metabolite and compartment IDs. Next, reactions connected to metabolites in the shared external compartment are gathered. For each reaction, nodes are created for the external metabolites and connected to the corresponding community members node.

Next, the newly created network needs to be arranged. This is achieved by a tailored layout algorithm, which places the metabolites and organisms in four concentric circles (Figure 1A: step 2): Going inwards, the first and largest circle contains all the external metabolites that are only connected to a single community member. The second circle contains the community member nodes, evenly spaced. The third circle is reserved for metabolites connected to exactly two members. Such metabolites are instead placed between two member nodes, if they are adjacent in the layout, which is encouraged by the algorithm. The inner circle contains all remaining metabolites, with three or more interaction partners. This layout algorithm can be applied to all networks generated by ScyNet via the menu.

In addition, ScyNet allows styling of the network according to network state. For this, a flux vector can be imported and ScyNet will colour the edges accordingly (see an example at Figure 1A, step 3). Networks can be stylized according to reaction fluxes of a single state by providing a flux vector as a tab-separated file containing two columns: ‘reaction_id’ and ‘flux’. Based on the reaction fluxes, all edge arrows and colours are set to represent the flux state. Reactions that do not carry flux are dropped from the visualization. Similarly, flux ranges, as in the output of FVA can be visualized as well. For this, a tab-separated file with three columns (‘reaction_id’, ‘min_flux’ and ‘max_flux’) is required. In addition to the indication of reaction directionality by colour, ScyNet can visualize flux values by setting the edge width proportionally.

All of the simplified networks produced by ScyNet can be further structured and stylized using methods provided by Cytoscape and its apps. However, when flux data are present, the networks can be focused to only visualize cross-feeding interactions, which are interactions of a community member producing a metabolite that is taken up by a different community member. This definition allows more than the required two community members to be part of cross-feeding interactions of the same metabolite. Further, cross-feeding interactions are considered regardless of the presence of the target metabolite in the medium. A visualization of the cross-feeding network can be achieved via the toggle cross-feeding function, hiding all reactions and metabolites not involved in cross-feeding (Figure 1A: step 4). The same function can be used to reveal the hidden components of the network again.

## 3 Results

For testing ScyNet we chose a microbial community of cystic fibrosis airways, illustrating a typical visualization use case of a small microbial community metabolic model. The microbial community was modelled using AGORA 1.02 models (Magnúsdóttir *et al.* 2017) as representatives for each genus, as done by Henson *et al.* (2019). The community consists of 17 members, of which only metabolic models of four key members (*Prevotella*, *Pseudomonas*, *Staphylococcus*, *Streptococcus*) were visualized.

The single species models were merged into a community metabolic model using PyCoMo. Flux ranges of reactions were calculated with FVA (90% of maximum growth-rate, equal abundance of members) and written to a tab-separated file for input in ScyNet. The total community metabolic model of the 4 members has 4787 metabolites and 5636 reactions (Figure 1A: step 1). In the simplified network created by Scynet these numbers are reduced to 234 metabolites and 568 reactions (Figure 1A: step 2).

After applying the reaction flux ranges and removing infeasible reactions, the model contains only 194 metabolites and 470 reactions (Figure 1A: step 3). By reducing the visualization to only cross-fed metabolites the network shrinks to 68 nodes and 222 edges.

Henson *et al.* (2019) analysed 16S rRNA data of 75 sputum samples of adult cystic fibrosis patients. This data showed that the community members can be present in varying compositions, which not only differ in flux amount, but also in activity and direction of exchange reactions, according to simulations of the metabolic models. We reproduced the visualization in figure 8D, showing predicted cross-feeding fluxes in the context of communities with high *Pseudomonas* abundance as an example (Figure 1B). An exact replication of the flux vector is not possible, as it is the average of the flux vectors across 500 simulations with randomly permuted reaction bounds. Instead, the flux vector was derived manually from the results of Henson *et al.* (2019), putting a flux of 1 for exchange reactions shown in figure 8D and omitting all others.

In the scenario of high abundance of *Pseudomonas*, alanine is predicted to be produced by *Staphylococcus*, *Prevotella* and *Streptococcus* and taken up by *Pseudomonas*. However at low abundance of *Pseudomonas* and increased abundance of *Streptococcus* the results of Henson *et al.* (2019) predict *Staphylococcus* to take up alanine produced solely by *Streptococcus*. This example shows that some cross-feeding interactions can be realized in both directions, with the organisms involved switching between their roles as producer and consumer.

In fact, flux ranges predicted by FVA show that the majority of external reactions which are part of cross-feeding interactions can carry flux in both directions (77.5%), meaning that many interactions themselves are reversible. While the reactions that facilitate the exchange of metabolites with the shared external compartment are reversible and unconstrained, less than half of them are bidirectionally active in the solution space calculated by FVA. The fraction of reactions which can carry flux in both directions is significantly lower in external reactions not involved in cross-feeding (8.1%), compared to external reactions of cross-fed metabolites (*P*-value ≪.05), suggesting high flexibility in the directionality of cross-feeding interactions in this community.

## 4 Conclusion

ScyNet enables comprehensible visualization of community metabolic models and the interactions of their members. The reduction in complexity makes the visualizations suited for communication of results, as shown in the reproduced figure of the four member cystic fibrosis airways microbial community by Henson *et al.* (2019). The visualization process takes less than 5 minutes on a desktop computer for the given example, including the time needed to generate the community metabolic model from the individual AGORA reconstructions. Thus, ScyNet can be readily applied during the construction, curation and analysis of community metabolic models.

ScyNet also allows to visualize different metabolic states of a community, e.g. for comparing communities under distinct conditions, different abundance profiles, growth on distinct media or showing FBA and FVA solutions. The contextualization of the reduced network could be extended by OMICs data integration as achieved by IDARE2 Pfau *et al.* (2021), but focused on the community level.

Most community metabolic modelling applications are done in small to medium communities of less than 20 members (Heinken *et al.* 2021). Natural communities often consist of more than 100 different taxa. Few studies have already attempted to model such complex communities with metabolic modelling and it is to be expected that more work will follow. While ScyNet drastically reduces the complexity of community metabolic networks, the amount of external metabolites and reactions grows with the size of the community (Ibrahim *et al.* 2021). The larger the network is the less informative will be its visualization.

To ensure that the represented network can be read at one glance, further reduction is necessary. An example is the selection of the five most frequent cross-feeding metabolites by Henson *et al.* (2019). Alternatively, modularization and abstraction can be used to deal with larger communities. Such coarse-graining could be achieved by combining organisms to functional guilds or grouping metabolites in categories.

Despite the challenge of highly complex and information-rich community metabolic models, ScyNet allows for quick visualization, analysis and communication of interactions between community members.
